# A fast approach to global alignment of protein-protein interaction networks

**DOI:** 10.1186/1756-0500-6-35

**Published:** 2013-01-31

**Authors:** Giorgos Kollias, Madan Sathe, Shahin Mohammadi, Ananth Grama

**Affiliations:** 1 Department of Computer Science, Purdue University, 305 N. University Street, West Lafayette, IN 47907, USA; 2 Department of Mathematics and Computer Science, University of Basel, Klingelbergstrasse 50, 4056 Basel, Switzerland; 3 Center for Science of Information, West Lafayette, IN 47907, USA

## Abstract

**Background:**

Global network alignment has been proposed as an effective tool for computing functional orthology. Commonly used global alignment techniques such as IsoRank rely on a two-step process: the first step is an iterative diffusion-based approach for assigning similarity scores to *all* possible node pairs (matchings); the second step applies a maximum-weight bipartite matching algorithm to this similarity score matrix to identify orthologous node pairs. While demonstrably successful in identifying orthologies beyond those based on sequences, this two-step process is computationally expensive. Recent work on computation of node-pair similarity matrices has demonstrated that the computational cost of the first step can be significantly reduced. The use of these accelerated methods renders the bipartite matching step as the dominant computational cost. This motivates a critical assessment of the tradeoffs of computational cost and solution quality (matching quality, topological matches, and biological significance) associated with the bipartite matching step. In this paper we utilize the state-of-the-art core diffusion-based step in IsoRank for similarity matrix computation, and couple it with two heuristic bipartite matching algorithms – a matrix-based greedy approach, and a tunable, adaptive, auction-based matching algorithm developed by us. We then compare our implementations against the performance and quality characteristics of the solution produced by the reference IsoRank binary, which also implements an optimal matching algorithm.

**Results:**

Using heuristic matching algorithms in the IsoRank pipeline exhibits dramatic speedup improvements; typically ×30 times faster for the total alignment process in most cases of interest. More surprisingly, these improvements in compute times are typically accompanied by *better* or *comparable topological and biological quality* for the network alignments generated. These measures are quantified by the number of conserved edges in the alignment graph, the percentage of enriched components, and the total number of covered Gene Ontology (GO) terms.

**Conclusions:**

We have demonstrated significant reductions in global network alignment computation times by coupling heuristic bipartite matching methods with the similarity scoring step of the IsoRank procedure. Our heuristic matching techniques maintain comparable – if not better – quality in resulting alignments. A consequence of our work is that network-alignment based orthologies can be computed within minutes (as compared to hours) on typical protein interaction networks, enabling a more comprehensive tuning of alignment parameters for refined orthologies.

## Background

The description of a cell as a collection of pathways of interacting biochemical components is fundamental to a systems view of biological processes. Data relating to regulatory, metabolic, and signaling interactions, is systematically, and naturally encoded into networks [1]. The diversity of species, cellular processes, abstractions, and volume of interaction data generated from high-throughput techniques strongly motivates development of effective and efficient analysis algorithms. Over the past two decades, significant progress has been made on algorithms for identifying conserved components, discriminating components, modularity, clustering, and alignment, of sequences, sets, and special graph structures (trees, DAGs). However, solving these problems for general large sparse graphs, while providing sound statistical basis for results, remains a topic of significant ongoing investigation. Effective solutions to these problems must leverage properties of specific datasets to deliver desirable performance.

In this paper, we focus on the problem of *network alignment*. This problem aims to quantify the similarity of two given graphs, resulting in the mapping of nodes from one graph to the other, along with the induced edge mapping. As a specific instance of this problem, in protein interaction (PPI) networks, physically interacting proteins are represented as edge-connected nodes. Identifying (topological) regions of similarity between networks of different species reveals insights into the functional organization and coherence of sub-networks. Specifically, if connected sub-graphs are conserved across species, they *likely* correspond to shared function across and within sub-graphs. This can be used for annotating proteins (by mapping annotations across species), inferring missing interactions, and drawing functional orthologies.

Network alignments can be derived from local or global measures of cost. In *local* network alignment (LNA) [2-4], local scoring functions reward potentially small subgraph matches. In PPIs, it follows that a node (protein) may potentially participate in many mappings. In contrast, *global* network alignment (GNA) [5-7] relies on a cost function defined over entire networks. This implies that in PPIs, a single protein from one network is mapped to a single protein in the other.

Proposed approaches to global alignment typically proceed in two steps: 

•In the first step, a similarity score matrix *X* is constructed, where element *x*_*ij*_ denotes the similarity of node *i* in the first graph to node *j* in the second graph.

•In the second step, a node matching algorithm selects pairs of nodes, one from each network, optimizing an aggregate similarity score measure using matrix *X*.

The similarity of two nodes is determined by the similarity of their interaction profiles, also called topological similarity, and the inherent similarity of the nodes. The latter notion of inherent similarity is introduced in by Singh et al. [6,7], as elemental (or node) similarity. Elemental similarity scores supplement topological similarity, and rely on node labels or attributes. As an example, for protein pairs, sequence similarity scores, independently computed by BLAST, can be used for elemental similarity. The method of Singh et al. [6,7], called IsoRank, uses a notion of topological similarity based on an iterative diffusion process for computing matrix *X*. In this method, the similarity of a pair of nodes is iteratively determined by the similarity of their neighbors. The topological similarity thus computed is accumulated with the elemental similarity at each iterative step and the process is run to convergence to yield matrix similarity matrix *X*.

In the second step of the method, best-matching pairs of nodes are identified. The matrix *X* is viewed as a weighted bipartite graph *G*=(*V*_*A*_,*V*_*B*_,*E*), where node *i* of the first graph represents a vertex in *V*_*A*_, node *j* of the second graph illustrates a vertex in *V*_*B*_ and matrix entry *x*_*ij*_ represents a weighted edge between the two nodes. Bipartite graph matching algorithms can be applied to obtain a set of edges *M*, *M*⊆*E*, such that no pair of edges in *M* are incident on/to the same vertex. Furthermore, the sum over the weighted edges in *M* is maximized (IsoRank [6,7], NetAlignBP [8], H-GRAAL [9]).

In IsoRank, the computation of matrix *X* represents the dominant cost. However, algorithmic improvements to this step have resulted in significant reductions in the cost of similarity matrix computation [10], particularly in cases with a small set of dominant, elemental similarity components. This has resulted in the second, bipartite matching step now becoming the performance bottleneck, and consequently, the focus of performance improvements.

In this paper, we critically examine the need for optimal bipartite matching, suitable heuristic algorithms for bipartite matching, the associated improvements in overall runtime, and their implications for quality of solution, both in terms of biological implications and topological matches.

Specifically, for a series of PPI network pairs, we experiment with our customized implementation of the two stage pipeline: we utilize the IsoRank scoring matrix calculation as the first stage, followed by the application of the matrix-based, greedy, and adaptive, auction-based matching algorithms. These two methods generate two alignments for each input network pair – (mat3_greedy and mat3_auction results). We then run, for the same input networks and parameters, the reference native binary implementation of IsoRank made available by Singh et al.[6], who report that their software applies the greedy and Hungarian algorithms to the scoring matrix *X*, and produces iso_greedy and iso_hungarian alignments.

*Our key result is an improvement in overall runtime of over one order of magnitude – typically an acceleration of* ×30*for almost all tested PPI network pairs – with comparable, and in some cases superior results in terms of topological and biological quality (* mat3_* *versus* iso_* *computations).* This represents a reduction in runtime for typical cases of PPI networks from hours to minutes – thus enabling a more comprehensive exploration of the parameter space for extracting desirable orthologies.

## Results and discussion

We report on the computational cost and quality characteristics of our mat3_* alignments in comparison with the ones generated by the reference native binary implementation of the IsoRank (iso_* results). The set of PPI networks, sequence similarity of their corresponding proteins, and the executable files for IsoRank [6,11] were obtained through the IsoRank public website [11]. PPI networks in this dataset, the list of which is provided in Table 1, were collected from publicly available databases, such as BioGRID and DIP, as well as the datasets of Stelzl and Vidal. Associated sequence similarities were generated by using BLAST on the sequences retrieved from Ensembl. We use *α*=0.80 for fixed number of iterations (20) in all experiments.

**Table 1:** PPI network data

**Species**	**Dataset name**	**| *V *|**	**| *E *|**
fruitfly(*D. melanogaster*)	dmela	7518	25830
bacterium(*E. coli*)	ecoli	1821	6849
human(*H. sapiens*)	hsapi	9633	36386
yeast(*S. cerevisiae*)	scere	5499	31898

Species for which PPI network data is used in our experiments.

### Computational performance

**Figure 1:**
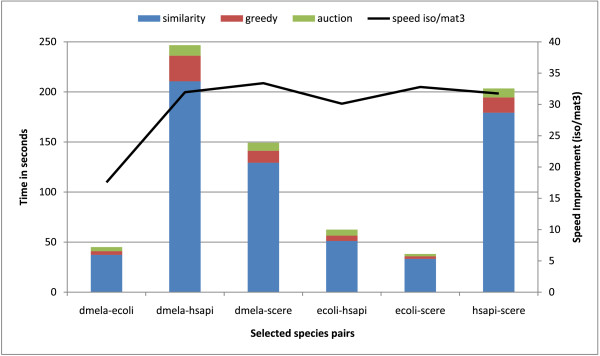
**Timing results.** Timing results for extracting all four types of matches of Figure 4. Times for obtaining mat3_* matches are represented as bars with colors identifying the relative contribution of each of the three algorithmic blocks (mat3, greedy, auction). The black line gives the corresponding speedup (as read on the right vertical axis) in generating our mat3_* matches relative to iso_* ones.

Computation times for mat3_* and iso_* implementations are plotted in Figure 1.

Two important observations can be made: 

•Our mat3_* matches are generated approximately 30 times faster than iso_*, for almost all pairs of networks. For the largest datasets this roughly translates to less than 5 minutes in our case (compared to iso_*’s 2 hours of processing time).

•The adaptive auction algorithm is a key element in this performance improvement, as opposed to the Hungarian algorithm — assuming IsoRank (constructing *X*) and greedy matching implementations are of comparable performance. Please note that we have no way of benchmarking these separately, since these times are not reported by the reference implementation of Singh et al. One may argue that our implementations of similarity matrix computations are much faster than corresponding implementations of Singh et al. While this is unlikely, even if this were to be the case, our significant performance improvements over the most widely distributed implementation of IsoRank represent the core of our contributions.

We also note that, especially for the largest pair of networks, our adaptive auction algorithm is the most efficient method for extracting matching pairs from similarity score matrices. Specifically, the only timing information that is available after running the native binary from [11] is the overall running time, which includes three separate components – (i) *their* similarity matrix construction phase, (ii) *their* Hungarian algorithm run, and (iii) *their* greedy approach. These three times cannot be separated. However *we can measure, separately,* the times for (i) *our* similarity matrix construction phase, (ii) *our* auction matching run, and (iii) *our* greedy approach run. Only the implementation of the second part, i.e, their Hungarian versus our auction algorithm, are fundamentally different. The corresponding similarity computations in the first phase and the greedy matching algorithm rely on similar algorithms. This suggests that the performance gain that we observe in the overall running time from the three parts in both cases (roughly ×30 speedup) can be primarily attributed to the performance difference between *their* Hungarian and *our* auction algorithm implementations.

In terms of the number of operations, the complexity of the Hungarian algorithm is O(|V|(|E|+|V|log|V|)). This is in comparison to the worst case complexity of an *ϵ*-scaling auction algorithm, which is given by O(|V||E|log(|V|C)) for integer weights (with *C*= max*ij*|*x*_*ij*_| and *x*_*ij*_ the similarity scores). Here |*V*| is the maximum number of vertices between the two graphs to be aligned and |*E*| is the number of entries in *X*. Experimental results suggest that the *worst case* runtime for the auction algorithm is rare. Other causes for the speed improvements include suboptimal data structures in similarity matrix computations in the binary code, or an unoptimized Hungarian implementation. These are hard to decipher from the binary – in either case, our software yields significant overall acceleration of the state-of-the-art approach from Singh et al. for the global alignment problem.

### The alignment graph and its assessment

#### Topological perspective

**Figure 2:**
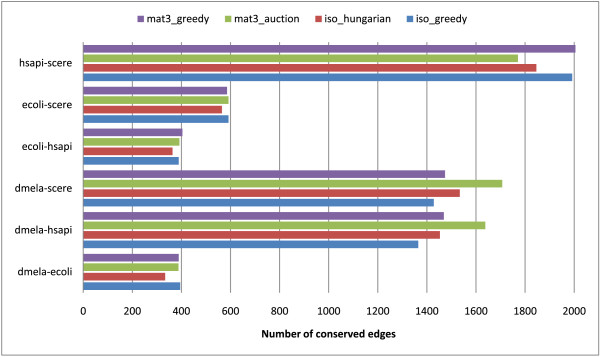
**Number of conserved edges in the alignment graphs.** Number of conserved edges in the alignment graphs for all combinations of species pairs and computation methods.

In Figure 2, the number of conserved edges in the alignment graph is reported. Our proposed mat3_auction approach outperforms the other methods in at least 2 out of the 6 cases – more conserved edges imply better alignments, as described in the *Methods* Section – and that the mat3_* matchings are superior to the iso_* ones (in 5 out of 6 cases, and on average). However there is no clear “winner”, i.e. a single method that is the best for all test cases.

#### Biological perspective

To assess the functional coherence of computed alignments, we report on their sensitivity and specificity (please see *Methods* Section for more details). Table 2 summarizes the corresponding statistics. We then provide a detailed analysis for a subset of the top-ranked components with co-enriched terms, where a functional term is enriched in the respective subgraphs of both input networks (from different species).

**Table 2:** Biological validation of alignment graphs

**(a) D.melanogaster vs S.cerevisiae**				
	**Fly**		**Yeast**	
**Method**	**TPR**	**TNR**	**TPR**	**TNR**
iso_greedy	535	17.00%	324	43.00%
iso_hungarian	457	15.20%	1066	44.00%
mat3_auction	490	15.40%	1132	38.30%
mat3_greedy	448	17.00%	1102	44.00%
**(b) D.melanogaster vs H.sapiens**				
	**Fly**		**Human**	
**Method**	**TPR**	**TNR**	**TPR**	**TNR**
iso_greedy	1067	30.10%	191	9.00%
iso_hungarian	974	31.50%	671	14.30%
mat3_auction	1020	27.00%	519	12.50%
mat3_greedy	1029	30.80%	670	17.00%
**(c) D.melanogaster vs E.coli**				
	**Fly**		**Bacterium**	
**Method**	**TPR**	**TNR**	**TPR**	**TNR**
iso_greedy	41	11.63%	66	16.60%
iso_hungarian	60	10.38%	175	29.35%
mat3_auction	90	9.80%	236	32.00%
mat3_greedy	56	10.73%	235	32.58%
**(d) H.sapiens vs S.cerevisiae**				
	**Human**		**Yeast**	
**Method**	**TPR**	**TNR**	**TPR**	**TNR**
iso_greedy	971	31.74%	406	17.97%
iso_hungarian	1010	31.27%	1063	41.76%
mat3_auction	1064	28.40%	1083	40.16%
mat3_greedy	989	33.33%	1095	46.14%
**(e) S.cerevisiae vs E.coli**				
	**Yeast**		**Bacterium**	
**Method**	**TPR**	**TNR**	**TPR**	**TNR**
iso_greedy	419	46.10%	63	15.00%
iso_hungarian	359	46.60%	231	38.24%
mat3_auction	400	40.72%	274	35.75%
mat3_greedy	378	44.22%	289	35.37%
**(f) E.coli vs H.sapiens**				
	**Bacterium**		**Human**	
**Method**	**TPR**	**TNR**	**TPR**	**TNR**
iso_greedy	52	16.77%	472	29.56%
iso_hungarian	158	35.63%	386	28.40%
mat3_auction	252	32.96%	557	34.80%
mat3_greedy	219	36.00%	444	29.73%

Biological validation of alignment graphs based on the method of Kalaev et al. [21]. The TPR column contains the total number of GO terms covered in the biological process branch, and the TNR column represents the percent of components that were enriched in each species.

#### Sensitivity

In terms of overall sensitivity (average of true-positive rate (TPR) in the pair of aligned species), mat3_auction shows superior performance, except in aligning human-versus-fly, where mat3_greedy outperforms the mat3_auction method. It is notable here that sensitivity is *not* comparable across different tables, since the total number of expected enriched terms is a function of evolutionary distance among species pairs that are being aligned. Species that have diverged more recently are more probable to have common pathways, which results in higher number of identified terms. However, for a fixed pair of species, which defines a unique functional space, we can use sensitivity as a measure to compare different methods.

#### Specificity

In terms of average specificity, we observe more diversity among different methods. Surprisingly, mat3_greedy is the top-ranked method in 4 out of 6 experiments, except in aligning yeast-versus-bacterium and bacterium-versus-human, for which iso_hungarian and mat3_auction perform better, respectively. These results suggest that the well-known Hungarian algorithm for maximum weighted bipartite matching does not necessarily enhance the biological quality of the results. One possible explanation for this phenomena is the *over-fitting problem*. The objective function for the matching phase is defined over the set of pairwise similarity scores for the nodes in different graphs, which itself is computed using both sequence and topological similarities. We note that both of the initial scores – sequence similarity scores computed using BLAST, and aggregated scores computed using IsoRank, are inherently noisy and over-fitting a model (matching) on them can potentially decrease the performance of the results.

#### Examples of highly enriched components

**Figure 3:**
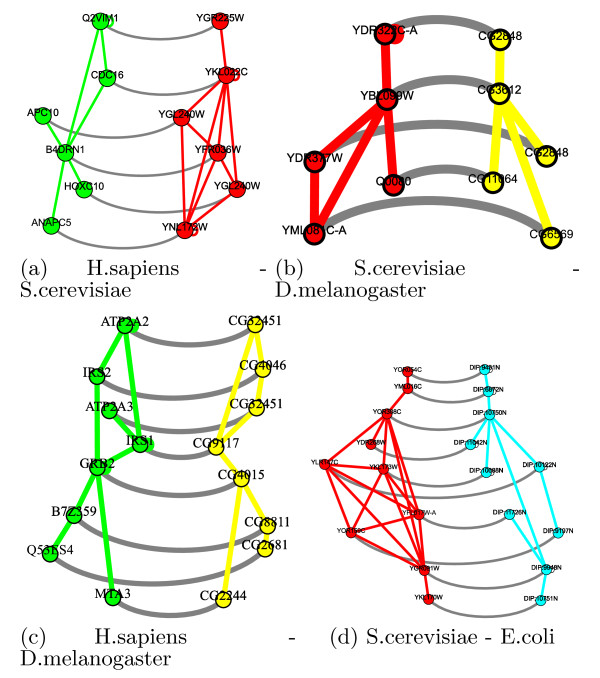
**Conserved components identified by the mat3_auction method.** Conserved components identified by the mat3_auction method. Each component is coherently annotated with specific branch of the biological process. (**a**) Peroxisome organization. (**b**) RNA splicing. (**c**) Histone modification. (**d**) Ribosome biogenesis.

We also evaluate the performance of the mat3_auction method by extracting components that are highly enriched in *both* species with respect to a *unique* GO term. We manually curated the components identified by mat3_auction and selected four significant components for our case study, spanning four different species pairs. These components, which are shown in Figure 3, reveal that there is a strong correlation between structural conservation and functional similarity, which has been faithfully recovered by mat3_auction. Most of these components have more than one co-enriched GO term, but interestingly enough, these terms are coherent in the sense that they describe the same function in more or less detail. For example, component 3(b) is annotated with *RNA processing*, which is a generalization of the term *nuclear mRNA splicing via spliceosome*, another enriched term in 3(b). Similarly, component 3(c) is enriched with both *histone acetylation* and *histone modification*. The first term is clearly a refinement of the type of modification.

These functionally similar modules exhibit closely related structure. In most cases, the smaller subgraph and all its edges can be perfectly embedded in the other species. The set of missed interactions can be explained by either evolutionary divergence or the quality of the input dataset.

From an evolutionary standpoint, one can argue that the set of orthologous genes in the pair of species could have diverged and either gained or lost specific functions. These functional adaptations are reflected in the pattern of protein-protein interactions by deleting or inserting partnerships. On the other hand, an easier argument can be made based on the quality and the coverage of proteome, noting that different PPI interactions have been predicted in different labs for each species. Some of these species are well-studied and there are more high-quality interactions available for them. As is evident from Figures 3(a) and 3(d), most of the missed edges reside in yeast, which can simply be explained by heterogeneity in the quality of initial PPI networks.

## Conclusions

Our results show that the IsoRank-based method for computing similarity scores between nodes of two PPI networks, coupled with a fast, adaptive, auction-based implementation and a matrix-based greedy algorithm for extracting matching proteins, yields an efficient and effective algorithm for global alignment. The method yields over one order of magnitude improvement in computation time – typically ×30 times faster for the total alignment process in most cases of interest – with comparable or superior topological and biological quality of the results. Using out method, alignments can be computed within minutes (less than 5 minutes for typical alignments), as compared to hours, using previously used methods (of the order of 130 minutes for the largest input configurations). This enables users to tune alignment parameters much better and extract superior alignments.

## Methods

### Ranking node pairs in IsoRank

We use the similarity matrix construction step in IsoRank in all alignment methods. This method is based on an analogy between the network alignment problem and that of identifying “reputed” nodes in a single network – also sometimes called the page- or node-ranking problem. Perhaps, the most commonly used measure for the rank of a node in a single given network can be recursively defined as follows: “a node is important if it is linked to other important nodes” [12]. Extending this definition to our node similarity problem, we arrive at the following definition: “two nodes are (topologically) similar if they are linked to other (topologically) similar node pairs” [6,7,13]. IsoRank effectively implements this notion of “recursive node similarity”. Note that this perspective is not new – it has been exploited in application areas like automated image captioning [14] and synonym extraction [15].

We initiate a more formal discussion by introducing necessary notation. Given a graph *G*_*A*_=(*V*_*A*_,*E*_*A*_), *V*_*A*_ and *E*_*A*_ denote the vertices and edges of *G*_*A*_ respectively, and *n*_*A*_=|*V*_*A*_|. Its adjacency matrix *A* has elements *a*_*ij*_=1 iff edge (*i*,*j*)∈*E*, and *a*_*ij*_=0 otherwise. Clearly, *A* is symmetric for an undirected graph. Matrix Ã is the normalized version of the matrix *A*^*T*^; formally, ${\left(Ã\right)}_{\text{ij}}=a_{\text{ji}}/\overset{n_{A}}{\underset{i=1}{\sum }}a_{\text{ji}}$ for nonzero rows of *A* and zero otherwise. We denote by 1, the column vector of size *n*_*A*_ consisting of 1’s. Also, the *vec*(·) operator for stacking matrix columns into a vector (as well as its associated “inverse” *unvec*(·) operator for re-assembling the matrix) are used. Using these operators, *vec*(*AXB*)=(*B*^*T*^⊗*A*)*x*, holds, where ⊗ denotes the Kronecker product of two matrices.

In IsoRank, vertex similarity scores in (PPI) networks are computed by integrating both vertex attributes (similarity of protein sequences) and topological affinity (links to similar nodes). More specifically, in [6], Singh et al. introduce the following iterative procedure for computing similarity scores:

(1) $$x\leftarrow \mathrm{\alpha Ã}⊗\tilde{B}x+(1-\alpha )\mathrm{h.}$$

Here *x*=*vec*(*X*) is the vector vertically stacking the columns of the similarity matrix *X* having as entries the similarity scores *x*_*ij*_. *h*=*vec*(*H*), similarly stacks elements *h*_*ij*_ of matrix *H*, i.e., the elemental similarity scores between nodes *i*∈*V*_*B*_ and *j*∈*V*_*A*_. Vectors *x* and *h* are normalized to unity. Successive iterates scale topological similarity and elemental similarity of nodes by factors *α*≤1 and 1−*α*, respectively. In the context of PPI networks, vector *h* encodes protein sequence similarity scores in particular, and protein interaction networks *G*_*A*_ and *G*_*B*_ are assumed to be undirected. By “unvec”ing (1), we obtain:

(2) $$X\leftarrow \alpha \tilde{B}X{Ã}^{T}+(1-\alpha )\mathrm{H.}$$

### Matching algorithms

As a first step in identifying similar subgraphs in the two networks, IsoRank computes a |*V*_*A*_|-by- |*V*_*B*_| similarity score matrix *X*. We assume without loss of generality that |*V*_*A*_|≤|*V*_*B*_|. The matrix can be viewed as encoding a weighted bipartite graph *G*=(*V*_*A*_,*V*_*B*_,*E*,*w*), where *V*_*A*_∩*V*_*B*_=*∅*, *E*⊆*V*_*A*_×*V*_*B*_, and the weight function $w:E\to R^{\geq 0}$. Each row represents a vertex in *V*_*A*_ and each column a vertex in *V*_*B*_. A non-zero entry in the matrix is interpreted as an edge between the row and column vertices. The numerical value *x*_*ij*_ of the matrix indicates the weight of the edge. A *matching* in the bipartite graph is defined as a subset *M*⊆*E* such that no pair of edges of *M* are incident on the same vertex. In a *maximal* matching, no edge can be added to *M* without violating the matching property. A *maximum* (cardinality) matching is a matching that contains the largest possible number of edges. Specifically, we are interested in finding a maximum matching with maximum weight. The weight of the matching is defined as $\underset{(i,j)\in M}{\sum }x_{\text{ij}}$; this typically translates to a large number of matching pairs with a high cumulative similarity score.

Various algorithms have been proposed for computing a weighted matching *M* for a given similarity matrix *X*. *Approximation* algorithms [16], which compute a maximal matching with a maximum weight, and maximum weighted matching algorithms [17,18], which return a maximum matching with a maximum weight, are all candidates for finding a suitable assignment. In our approach, we use the 1/2-approximation algorithm and propose a weighted matching implementation that is based on the principle of *auctions*. The main advantage of using an auction-based scheme is the so-called *ϵ*-scaling mechanism, using which the quality and convergence of the algorithm can be controlled.

#### *1/2*-approximation algorithm

This simple approximation algorithm can be described as follows: First, the weights of the edges are sorted in decreasing order. Then, the heaviest edge *e* is selected and deleted, together with the edges incident to its endpoints. This is repeated until the graph is empty. The worst case complexity of this sorting procedure is O(|E|log|E|). We implement the linear-time 1/2-approximation algorithm of Preis [16], of known time complexity O(|E|) (Algorithm 1), translating this graph-based description into matrix operations (matrix-based greedy algorithm): After selecting the element with maximum value *x*_*ij*_ from the similarity matrix *X*≠0, we report the matching of nodes *i* and *j*. We then zero the *i*^*th*^ row and the *j*^*th*^ column of *X*, and repeat the aforementioned step until *X* contains only zeros across one of its dimensions.

##### Algorithm 1 1/2-approximation algorithm for weighted matching

#### Auction algorithms

Auction algorithms [18] find the maximum weighted matching via an *auction*: in this analogy, *i*∈*V*_*A*_ is a person, *j*∈*V*_*B*_ is an object and *x*_*ij*_ is the benefit the buyer *i* obtains by acquiring object *j*. Each object *j* has an associated price *p*_*j*_, with the initial value zero.

##### Algorithm 2 Auction Algorithm

In an auction iteration, the bidding and assignment phase, and update of the price and of the value of *ϵ* are performed. In the bidding phase, an unassigned buyer *i* bids for the best object *j*_*i*_, i.e., the object *j*_*i*_, that has the maximal profit for buyer *i*. The bid is computed by subtracting the second-best profit *v*_*i*_ from the most valuable profit *u*_*i*_, i.e., *u*_*i*_−*v*_*i*_. The most valuable profit *u*_*i*_ for buyer *i* is defined as $\left\{x_{ij_{i}}-p_{j_{i}}\right\}$, while the second-best profit *v*_*i*_ is computed by $\underset{j\neq j_{i}}{\max}\{x_{\text{ij}}-p_{j}\}$. After the unassigned buyer has submitted the bid, the designated object is awarded to the bidder, yielding its potential previous owner unassigned. The price is calculated by updating the old price by the corresponding bid and by a small increment *ϵ*. It follows that the auction-based algorithm (see Algorithm 2 for a simplified description also assuming integer *x*_*ij*_) consists of four phases: the *initialization phase* (lines 1–7), the *bidding phase* (lines 9–12), the *assignment phase* (lines 13–15), and the *termination phase* (line 8).

The initial value of increment *ϵ* has significant impact on the computational cost of the auction algorithm. Ideally, the value of *ϵ* should be close to the optimal value of the price, since the number of iterations to find a matching will be small. In general, the computational worst case complexity of an *ϵ*-scaling auction algorithm is O(|V||E|log(|V|C)) (where *C* is defined in line 5 of Algorithm 2). Due to the pseudo-polynomial complexity, we embed an aggressive *ϵ*-scaling strategy into the auction-based implementation of Algorithm 2, resulting in our *adaptive auction algorithm*, which effectively splits the *ϵ*-scaling phase into multiple *ϵ*-scaling phases; its steps are detailed in Algorithm 3.

##### Algorithm 3 Adaptive Auction Algorithm

Here, the *ϵ*-value is initialized with a small value and adaptively increased relative to the overall progress of the matching. The basic idea behind the proposed heuristic is that in the inner iteration, at least *δ* buyers get assigned to an object, while *ϵ* pushes the price of the object to a large value. In general, the heuristic provides a maximal matching with a maximum weight. Since the matrix *X* is dense, a simple greedy approach is applied subsequently, in order to achieve a maximum matching. The post-processing method assigns an unmatched buyer to the first unmatched object from the object list, resulting in a maximum matching with maximum weight.

For a more detailed presentation of the auction algorithm we use, we refer readers to Sathe et al. [19], where a scalable distributed version of the algorithm is described. This formulation computes weighted matchings on sparse and dense bipartite graphs running on hundreds of compute nodes, while efficiently using multiple cores on each compute node.

### Numerical experiments

In all cases, the construction of the similarity matrix *X* is IsoRank-based. We implemented the iterative scheme of Equation (2) in Matlab (mat3_* cases — name mat3 is used to indicate a triple-matrix product kernel) and tested this part against equivalent codes in the netalign package [20], yielding exact agreement (within machine accuracy). The resulting matrix *X* of similarity scores is then input to (i) our matrix-based implementation (in Matlab) of the 1/2-approximation algorithm (hereafter referred to as *greedy*) to produce the mat3_greedy alignment and (ii) to the adaptive auction algorithm (in C) to generate the respective mat3_auction alignment.

**Figure 4:**
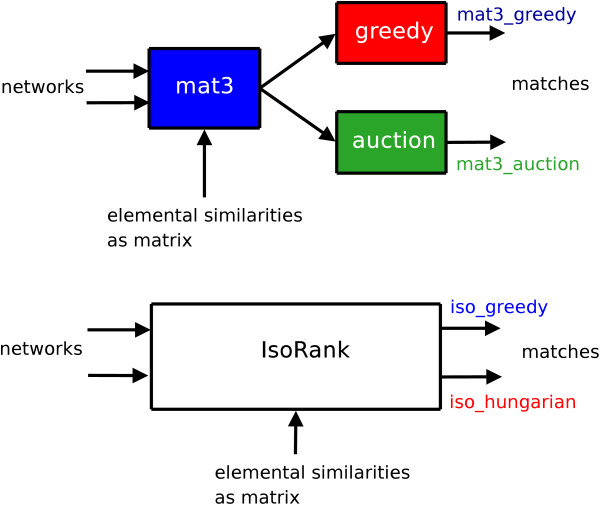
Algorithmic blocks Algorithmic blocks for the generation of the four types of matches (mat3_greedy, mat3_auction, iso_greedy, iso_hungarian) under comparison.

We then execute, for the same runtime parameters, the reference IsoRank binary for producing iso_greedy and iso_hungarian alignments and compare them against our approach (mat3_* alignments). Unfortunately the reference native code does not provide an option for generating either the similarity matrix *X*, or the timings for its computation. It internally uses the result of this first phase to extract the best matching node pairs, the second phase. The total timing results — for both phases — are reported together with the extracted matchings pairs in this case. Specifically, results from two matching algorithms are reported [6], namely their implementation of the greedy and Hungarian algorithms – thus justifying the selection of iso_greedy and iso_hungarian names for the complete alignment pipelines. It is understood that marked deviations could arise due to internal optimization-targeted realizations of the algorithmic ideas, however these are not readily accessible or reported. Figure 4 summarizes the algorithmic blocks driving the generations of four sets of matchings for each pair of PPI networks.

### The alignment graph and its assessment

Given a set of matching node pairs (one of the four mat3_*, iso_* possible alternatives in this context), we need to evaluate solution quality. The *alignment graph* is the auxiliary structure built to facilitate this task. Each node in the alignment graph is a matching node pair from the computed set of matches. Furthermore, if *m*_1_=(*i*_1_,*j*_1_) and *m*_2_=(*i*_2_,*j*_2_) in *V*_*A*_×*V*_*B*_ are two such matches, then *m*_1_ and *m*_2_ are connected by an edge iff *i*_1_ is connected with *i*_2_ and *j*_1_ with *j*_2_ in the input graphs *G*_*A*_ and *G*_*B*_. Essentially, the alignment graph identifies the link structures that remain invariant when replacing nodes of one graph with their matching counterparts in the other; it captures how our computed matchings between *nodes* preserves implicit/induced matchings between their incident *edges*.

#### Topological perspective

When analyzing the alignment graph of two networks, two measures for the topological evaluation of the computed match can be used: 

•The number of edges in the alignment graph (conserved edges). Note that the more the conserved edges – incident on our matching nodes in the two networks – the larger the percentage of “link structure” (edges at minimal or larger connected subgraphs) that could be put under direct mapping as well, i.e. *aligned*.

•The size of the connected components in the alignment graph (common connected subgraphs). These are “clusters” of *pairs* of matching nodes in the original graphs also conserving their link patterns.

The existence of many conserved edges clearly increases the probability of them being part of extensive connected subgraphs. However, it could also be the case that they are parts of larger numbers of connected subgraphs, all of moderate sizes. Comparative, connected subgraph statistics is the main focus of quality assessment from the biological point of view.

#### Biological perspective

Topological assessment of the alignment graph can uncover important characteristics of each alignment method. However, additional considerations must be taken into account to avoid growing components at the expense of over-generalizing them. This can negatively affect the specificity of predictions. For example, by analyzing the size of connected components, one can penalize against fragmenting functional modules. An alignment with many singletons (isolated pairs of nodes in the alignment graph) is less desirable than a larger connected component that can group a number of related nodes (and their corresponding proteins) together. On the other hand, by mixing functionally independent groups that share a small subset of genes, one can create larger components that are not functionally coherent. To remedy this problem, we adopt an approach similar to the one proposed by Kalaev et al. [21] to assess the functional coherence of each connected component in the alignment graph. In this approach, each connected component is treated as a computational prediction of a functionally related group of proteins and is cross-validated against the existing GO annotations as the actual set of functionally related genes. Given the gene ontology (GO) [22] annotations with respect to biological process (BP) for the member genes of each species, we compute the set of enriched GO terms within each connected component in the alignment graph using the GO::TermFinder tool [23]. We process each graph separately and, similar to Kalaev et al. [21], apply a threshold of 0.05 to extract the set of enriched GO terms. Finally, we define two complementary criteria to validate each alignment method – the fraction of enriched components (components with at least one enriched GO term) and the total number of covered GO terms in all components. The former captures specificity (true negative rate or TNR), while the latter captures the concept of sensitivity (true positive rate or TPR).

### Source code and datasets

All online material for this project is available at http://compbio.soihub.org/projects/fastalign/. This includes Matlab scripts, C code for the (more general, distributed version of the) auction algorithm, input datasets, and also data generated during intermediate phases of an actual run.

## Abbreviations

PPI: Protein Protein Interaction; DAG: Directed Acyclic Graph; GO: Gene Ontology; LNA: Local Network Alignment.

## Competing interests

The authors declare that they have no competing interest.

## Authors’ contributions

GK implemented and ran similarity matrix construction, greedy matching, ran Isorank binary code, designed and ran topological perspective experiments (except for auction-based ones), provided input for biological validation and drafted large parts of the manuscript. MS designed, implemented and ran auction-based matching methods, authored most of matching-related parts and provided Figures 1 and 2. SM authored the biological validation parts (methods and discussion) also providing Figure 3 and Table 2. AG provided guidance relative to the theoretical and practical aspects of the algorithms, initiated discussions on performance, validation and presentation issues and considerably improved the final form of the manuscript. All authors read and approved the final manuscript.
